# A Comparative Evaluation of Dapagliflozin and Sulfonylureas in Patients With Type 2 Diabetes Mellitus

**DOI:** 10.7759/cureus.115086

**Published:** 2026-08-24

**Authors:** Mohmed Sohel Shaikh, Suraj Tripathi, Chandra Bhanu Tripathi, Prashant M Parmar, Nilesh Patil, Hitesha H Trivedi

**Affiliations:** 1 Department of Pharmacology, Zydus Medical College and Hospital, Dahod, IND

**Keywords:** adverse drug reactions (adr), body weight, dapagliflozin, glimepiride, glycated hemoglobin (hba1c), glycemic control, metformin, type 2 diabetes mellitus (t2dm)

## Abstract

Introduction

Type 2 diabetes mellitus (T2DM) often requires combination therapy when metformin alone fails to achieve adequate glycemic control. Dapagliflozin, a sodium-glucose cotransporter-2 (SGLT2) inhibitor, and glimepiride, a sulfonylurea, are commonly prescribed as add-on therapies. This study compared their efficacy and safety in patients with inadequately controlled T2DM.

Methods

A nonrandomized prospective observational comparative study was conducted at a tertiary care teaching hospital between April and November 2024. A total of 170 adults with inadequately controlled T2DM receiving metformin (1000 mg) were allocated to receive either dapagliflozin (10 mg) (n=85) or glimepiride (2 mg) (n=85) and were followed for 24 weeks. Primary outcomes included changes in glycated hemoglobin (HbA1c), fasting blood sugar (FBS), and postprandial blood sugar (PPBS). Secondary outcomes included body weight, blood pressure, and adverse events.

Results

Both treatment groups demonstrated significant improvements in glycemic control over 24 weeks. At study completion, HbA1c was significantly lower in the dapagliflozin group than in the glimepiride group (7.60±0.64% vs. 8.00±0.68%; p=0.01). Glimepiride produced a greater reduction in FBS (101.82±20.65 vs. 115.98±15.09 mg/dL; p<0.001), while PPBS was comparable between groups (p=0.45). Dapagliflozin resulted in significant weight loss and reductions in systolic and diastolic blood pressure, whereas glimepiride was associated with weight gain. Overall, adverse events were more frequent with dapagliflozin, primarily polyuria and genital infections, while hypoglycemia and tremors occurred more frequently with glimepiride.

Conclusions

Both dapagliflozin and glimepiride were effective as add-on therapy to metformin in improving glycemic control. Dapagliflozin was associated with greater HbA1c reduction, weight loss, lower blood pressure, and a lower risk of hypoglycemia, whereas glimepiride achieved greater fasting glucose reduction. Treatment selection should be individualized based on patient characteristics, comorbidities, and therapeutic goals.

## Introduction

Type 2 diabetes mellitus (T2DM) is a chronic metabolic disorder characterized by persistent hyperglycemia resulting from impaired insulin secretion, insulin resistance, or a combination of both [1]. It accounts for approximately 90%-95% of all diabetes cases and represents one of the most significant global public health challenges because of its increasing prevalence and associated long-term complications [1]. Persistent hyperglycemia contributes to progressive damage of multiple organ systems, resulting in microvascular complications such as diabetic retinopathy, nephropathy, and neuropathy, as well as macrovascular complications including coronary artery disease, stroke, and peripheral arterial disease. These complications substantially increase morbidity, mortality, and healthcare expenditure [2,3].

According to the International Diabetes Federation (IDF), approximately 589 million adults were living with diabetes worldwide in 2025, and this number is projected to increase to 853 million by 2050 [4]. India bears one of the largest global burdens of diabetes, with prevalence increasing rapidly because of urbanization, aging, obesity, reduced physical activity, and unhealthy dietary habits. The growing burden of T2DM poses a major challenge to healthcare systems, particularly in low- and middle-income countries where early diagnosis and optimal long-term disease management remain difficult [4,5]. The progressive decline in pancreatic β-cell function, together with worsening insulin resistance, makes T2DM a chronic and progressive disease [1,6]. Consequently, glycemic control frequently deteriorates over time despite lifestyle modification and pharmacological intervention [6]. The United Kingdom Prospective Diabetes Study (UKPDS) demonstrated that every 1% reduction in glycated hemoglobin (HbA1c) significantly decreases the risk of microvascular complications and contributes to improved long-term clinical outcomes, emphasizing the importance of maintaining optimal glycemic control throughout the course of the disease [3]. Nevertheless, many patients eventually require combination pharmacotherapy because monotherapy becomes insufficient to achieve individualized glycemic targets [6].

Metformin remains the recommended first-line pharmacological treatment for T2DM because of its proven efficacy, favorable safety profile, low cost, minimal risk of hypoglycemia, and beneficial effects on insulin sensitivity [7]. However, the progressive nature of T2DM often necessitates the addition of a second antihyperglycemic agent when glycemic targets are not achieved with metformin alone [6-8]. Current treatment guidelines emphasize individualized therapy based on patient characteristics, cardiovascular risk, renal function, body weight, risk of hypoglycemia, and drug tolerability rather than glycemic efficacy alone [7]. Sulfonylureas continue to be among the most commonly prescribed second-line oral antidiabetic agents because of their potent glucose-lowering efficacy, rapid onset of action, and affordability [9]. Glimepiride, a second-generation sulfonylurea, lowers blood glucose by stimulating insulin secretion through closure of adenosine triphosphate (ATP)-sensitive potassium channels in pancreatic β-cells [10,11]. Although effective in reducing HbA1c, fasting plasma glucose, and postprandial glucose, sulfonylureas are frequently associated with hypoglycemia, weight gain, and reduced durability of glycemic control, which may limit their long-term clinical utility in certain patient populations [9-11].

Sodium-glucose cotransporter-2 (SGLT2) inhibitors have emerged as an important therapeutic class owing to their insulin-independent mechanism of action [12]. Dapagliflozin selectively inhibits glucose reabsorption in the proximal renal tubules, thereby promoting urinary glucose excretion and improving glycemic control [12]. Beyond glucose lowering, large cardiovascular outcome trials have demonstrated significant reductions in body weight, systolic blood pressure, and cardiovascular events [13,14]. In addition, SGLT2 inhibitors reduce hospitalization for heart failure and slow the progression of chronic kidney disease, making them an attractive therapeutic option for patients with T2DM and cardiovascular or renal comorbidities [15,16].

Although both glimepiride and dapagliflozin are widely used as add-on therapies to metformin, direct comparative evidence evaluating their efficacy and safety remains relatively limited, particularly in routine clinical practice and among Indian patients. Previous randomized trials have shown that dapagliflozin provides glycemic control comparable to sulfonylureas while offering additional advantages such as weight reduction, lower incidence of hypoglycemia, blood pressure reduction, and improved cardiovascular outcomes [17-19]. However, differences in ethnicity, clinical practice, socioeconomic factors, and patient characteristics necessitate further comparative evaluation in real-world settings [18,19].

The primary objective of this study was to compare the change in HbA1c from baseline to week 24 between patients receiving dapagliflozin or glimepiride as add-on therapy to metformin. We hypothesized that dapagliflozin would provide comparable glycemic control with additional benefits in body weight and a lower risk of hypoglycemia.

## Materials and methods

Study design

A prospective nonrandomized comparative observational cohort study was conducted at the Non-Communicable Disease (NCD) Clinic of the Department of Medicine, Zydus Medical College and Hospital, Dahod, Gujarat, India, between April 2024 and November 2024. The study compared the efficacy and safety of dapagliflozin (10 mg) and glimepiride (2 mg) as add-on therapy to metformin (1000 mg) in adult participants with inadequately controlled T2DM. Participants were managed according to routine clinical practice, and treatment allocation was determined solely by the treating physician rather than by the investigators. Thus, no intervention or randomization was performed by the study investigators.

The study protocol was approved by the Institutional Ethics Committee of Zydus Medical College and Hospital, Dahod, Gujarat (Approval No. ZMCH/IEC/021(06)-2024; approved on April 9, 2024). No participant was enrolled before Institutional Ethics Committee approval. Written informed consent was obtained from all participants before enrolment, and the study was conducted in accordance with the ethical principles of the Declaration of Helsinki [20].

Study population

Adult participants attending the Medicine Outpatient Department with a confirmed diagnosis of T2DM were screened for eligibility. Those fulfilling the inclusion and exclusion criteria (Table 1) and willing to participate were enrolled consecutively during the study period. A total of 170 participants were included in the study, with 85 receiving metformin (1000 mg) plus dapagliflozin (10 mg) (Group A) and 85 receiving metformin (1000 mg) plus glimepiride (2 mg) (Group B).

**Table 1:** Inclusion and exclusion factors in study population selection This table outlines the inclusion and exclusion criteria used to determine participant eligibility for the study, ensuring the enrollment of appropriate patients with type 2 diabetes mellitus while excluding individuals with conditions or treatments that could affect study outcomes or patient safety.

Inclusion Criteria	Exclusion Criteria
Adults aged 18-69 years	Type 1 diabetes mellitus
Diagnosed with type 2 diabetes mellitus (T2DM)	Pregnancy or lactation
HbA1c ≥6.5% at baseline	Severe hepatic impairment with Child Pugh Score between 10 and 15 points
Initiated on metformin plus dapagliflozin or metformin plus glimepiride as add-on therapy	Estimated glomerular filtration rate (eGFR) <45 mL/min/1.73 m²
Willing to provide written informed consent	Known hypersensitivity to metformin, dapagliflozin, or glimepiride
	Patients receiving insulin or other injectable antidiabetic therapy
	Active urinary tract infection or genital fungal infection at baseline
	Severe systemic illness or malignancy or with hypertension likely to interfere with study participation

The sample size was estimated using the prevalence of diabetes in Dahod district reported in the National Family Health Survey (NFHS-5), using the formula

$n = \frac{Z^{2}pq}{e^{2}}$

with a 95% confidence level and acceptable precision. The calculation yielded a minimum total sample size of approximately 150 participants. To account for potential loss to follow-up and to ensure adequate participant availability for the two study groups, 170 participants were enrolled, with 85 participants in each group.

Study procedure

Baseline demographic and clinical characteristics, including age, sex, body weight, blood pressure, fasting blood sugar (FBS), postprandial blood sugar (PPBS), and HbA1c, were recorded at enrolment. Patients received either dapagliflozin or glimepiride as add-on therapy to metformin according to the treating physician's routine clinical judgment. The investigators did not influence treatment selection or dosage adjustments, which were made solely by the treating physician whenever clinically indicated.

Participants were followed prospectively for 24 weeks, with scheduled evaluations at baseline, 12 weeks, and 24 weeks. The primary, secondary, and safety outcome measures are summarized in Table 2. During each visit, glycemic parameters, body weight, blood pressure, medication adherence, and adverse drug reactions were recorded using standardized case record forms.

**Table 2:** Primary, Secondary, and Safety Outcome Measures This table summarizes the predefined primary, secondary, and safety outcome measures evaluated during the 24-week follow-up period. Primary outcomes included changes in glycated hemoglobin (HbA1c), fasting blood sugar (FBS), and postprandial blood sugar (PPBS) from baseline to 24 weeks. Secondary outcomes included changes in body weight, systolic blood pressure (SBP), and diastolic blood pressure (DBP). Safety was assessed by documenting the incidence, type, and frequency of adverse drug reactions (ADRs) reported during follow-up.

Outcome Category	Outcome Measure	Assessment
Primary Outcome	Glycated haemoglobin (HbA1c)	Change in HbA1c (%) from baseline to 24 weeks
Primary Outcome	Fasting blood sugar (FBS)	Change in fasting blood glucose (mg/dL) from baseline to 24 weeks
Primary Outcome	Postprandial blood sugar (PPBS)	Change in postprandial blood glucose (mg/dL) from baseline to 24 weeks
Secondary Outcome	Body weight	Change in body weight (kg) from baseline to 24 weeks
Secondary Outcome	Systolic blood pressure (SBP)	Change in systolic blood pressure (mmHg) from baseline to 24 weeks
Secondary Outcome	Diastolic blood pressure (DBP)	Change in diastolic blood pressure (mmHg) from baseline to 24 weeks
Safety Outcome	Adverse drug reactions	Incidence, type, and frequency of adverse drug reactions reported during the 24-week follow-up period

Laboratory investigations were performed using standardized institutional laboratory procedures. HbA1c was measured using high-performance liquid chromatography (HPLC), while FBS and PPBS levels were determined using standard enzymatic methods. Blood pressure was measured using a calibrated sphygmomanometer after participants had rested for at least five minutes, and body weight was recorded using a calibrated digital weighing scale.

All adverse events occurring during follow-up were recorded. Events considered by the investigator to have a reasonable causal relationship with the study medication were classified as adverse drug reactions using the World Health Organization-Uppsala Monitoring Centre (WHO-UMC) causality assessment criteria. The severity of each event was graded as mild, moderate, or severe. Serious adverse events, treatment discontinuations due to adverse events, and their management were documented. Hypoglycemia was defined as a blood glucose level <70 mg/dL and classified as symptomatic, documented, or severe according to American Diabetes Association (ADA) criteria. Genital infections were diagnosed based on compatible clinical features with microbiological confirmation when indicated.

Statistical analysis

Data were entered into Microsoft Excel (Microsoft, Redmond, WA) and analyzed using IBM SPSS Statistics for Windows, Version 29.0 (IBM Corp., Armonk, NY, USA). Continuous variables were expressed as mean±standard deviation (SD), whereas categorical variables were presented as frequencies and percentages. Baseline characteristics were compared between the dapagliflozin and glimepiride groups using the independent Student's t-test for continuous variables and the Chi-square test or Fisher's exact test, as appropriate, for categorical variables. Changes in continuous outcome measures over the 24-week follow-up period within each treatment group were analyzed using repeated-measures analysis of variance (ANOVA). Between-group comparisons at each follow-up visit were performed using the independent Student's t-test. All statistical tests were two-sided, and a p value of <0.05 was considered statistically significant.

## Results

A total of 170 patients with T2DM were enrolled in this prospective comparative observational study. Of these, 85 patients received dapagliflozin plus metformin (Group A) and 85 received glimepiride plus metformin (Group B). All enrolled participants completed the 24-week follow-up, and complete outcome data were available for all participants; therefore, all 170 patients were included in the final analysis.

Baseline characteristics

The baseline demographic characteristics of the study participants are presented in Table 3. The mean age was 48.23±7.52 years in the dapagliflozin plus metformin group and 48.67±6.85 years in the glimepiride plus metformin group. The male-to-female distribution was 47:38 and 43:42 in Groups A and B, respectively. No statistically significant differences were observed between the two groups with respect to age or sex distribution (p>0.05), indicating comparable baseline demographic characteristics.

**Table 3:** Baseline demographic characteristics of the study participants The table summarizes the baseline demographic characteristics of participants in the dapagliflozin plus metformin and glimepiride plus metformin groups. No statistically significant differences were observed between the two groups at baseline. Values are presented as mean±standard deviation (SD) or number (percentage), as appropriate.

Characteristic	Dapagliflozin+Metformin (n=85)	Glimepiride+Metformin (n=85)
Age (years), mean±SD	48.23±7.52	48.67±6.85
Male, n (%)	47 (55.3)	43 (50.6)
Female, n (%)	38 (44.7)	42 (49.4)

**Figure 1:**
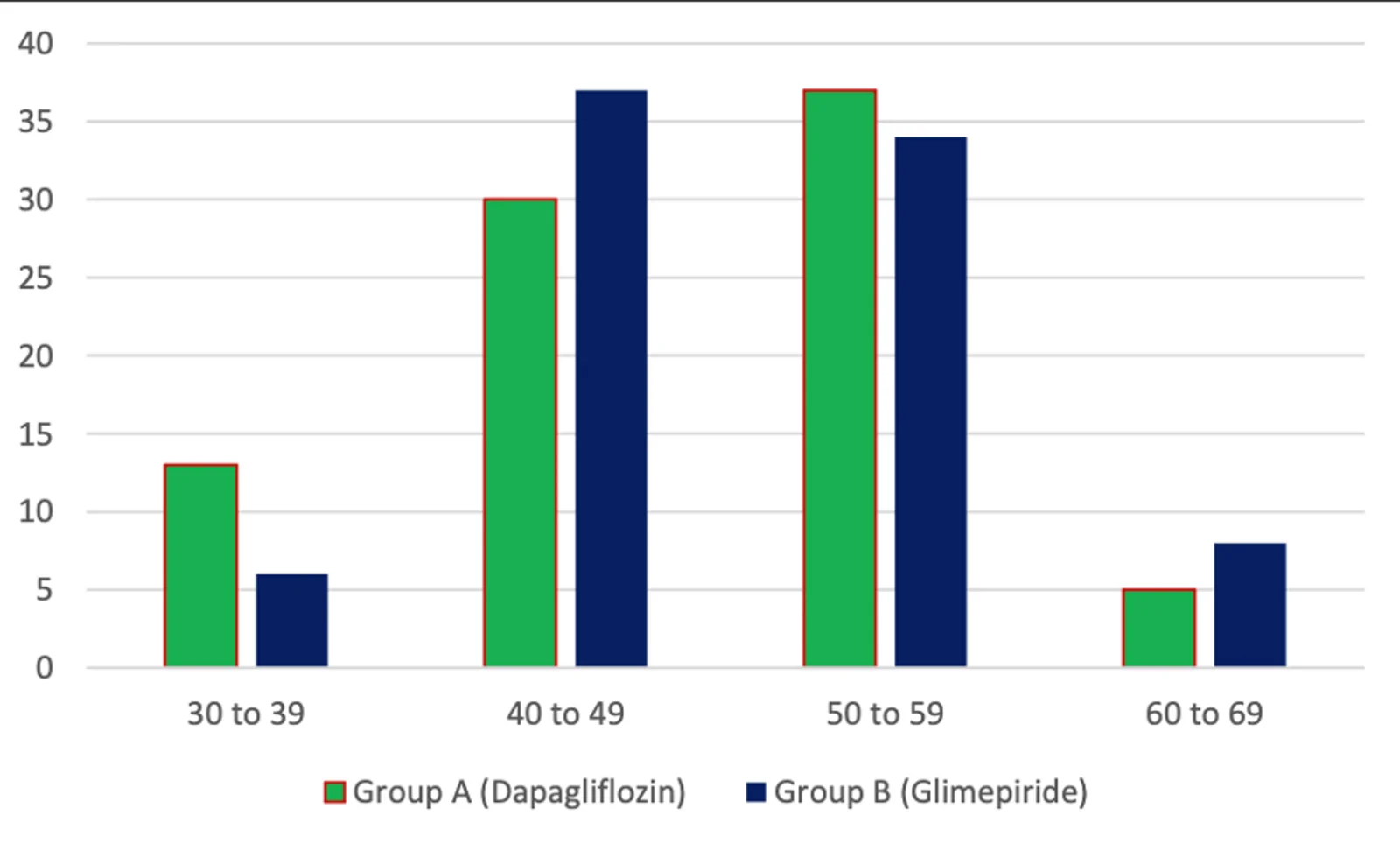
Age distribution of participants in the dapagliflozin plus metformin and glimepiride plus metformin groups The figure illustrates the distribution of study participants across different age groups in the dapagliflozin plus metformin (Group A) and glimepiride plus metformin (Group B) groups at baseline. The majority of participants in both groups were aged 40-59 years.

Figure 1 shows the age distribution of participants in the two treatment groups. 

Glycemic outcomes

Within-Group Comparison

Within-group changes in FBS and PPBS over the 24-week follow-up period are presented in Table 4. Both treatment groups demonstrated improvements in glycemic parameters during the study period.

In the dapagliflozin group, mean FBS decreased from 122.98±20.14 mg/dL at baseline to 119.05±11.77 mg/dL at 12 weeks and 115.98±15.09 mg/dL at 24 weeks. Although a downward trend was observed, the reduction in FBS did not reach statistical significance (p=0.0557). In contrast, PPBS decreased significantly from 182.65±10.29 mg/dL at baseline to 175.36±13.96 mg/dL at 12 weeks and 169.75±18.85 mg/dL at 24 weeks (p<0.001).

Similarly, in the glimepiride group, mean FBS decreased from 127.94±21.09 mg/dL at baseline to 120.03±10.57 mg/dL at 12 weeks and 101.82±20.65 mg/dL at 24 weeks, representing a statistically significant reduction (p<0.001). Mean PPBS also decreased significantly from 184.18±15.42 mg/dL at baseline to 175.59±31.09 mg/dL at 12 weeks and 174.60±16.37 mg/dL at 24 weeks (p=0.0199).

**Table 4:** Within-group changes in fasting blood sugar (FBS) and postprandial blood sugar (PPBS) over the 24-week follow-up period The table presents the within-group changes in fasting blood sugar (FBS) and postprandial blood sugar (PPBS) from baseline to 12 and 24 weeks in the dapagliflozin plus metformin and glimepiride plus metformin groups. Data are presented as mean±standard deviation (SD). P values represent within-group comparisons over the 24-week follow-up period. *Indicates statistical significance at p<0.05.

Group	Parameter	Baseline	12 Weeks	24 Weeks	p-value
Dapagliflozin (10 mg)+Metformin (1000 mg)	FBS (mg/dL)	122.98±20.14	119.05±11.77	115.98±15.09	0.0557
	PPBS (mg/dL)	182.65±10.29	175.36±13.96	169.75±18.85	<0.001*
Glimepiride (2 mg)+Metformin (1000 mg)	FBS (mg/dL)	127.94±21.09	120.03±10.57	101.82±20.65	<0.001*
	PPBS (mg/dL)	184.18±15.42	175.59±31.09	174.60±16.37	0.0199*

**Figure 2:**
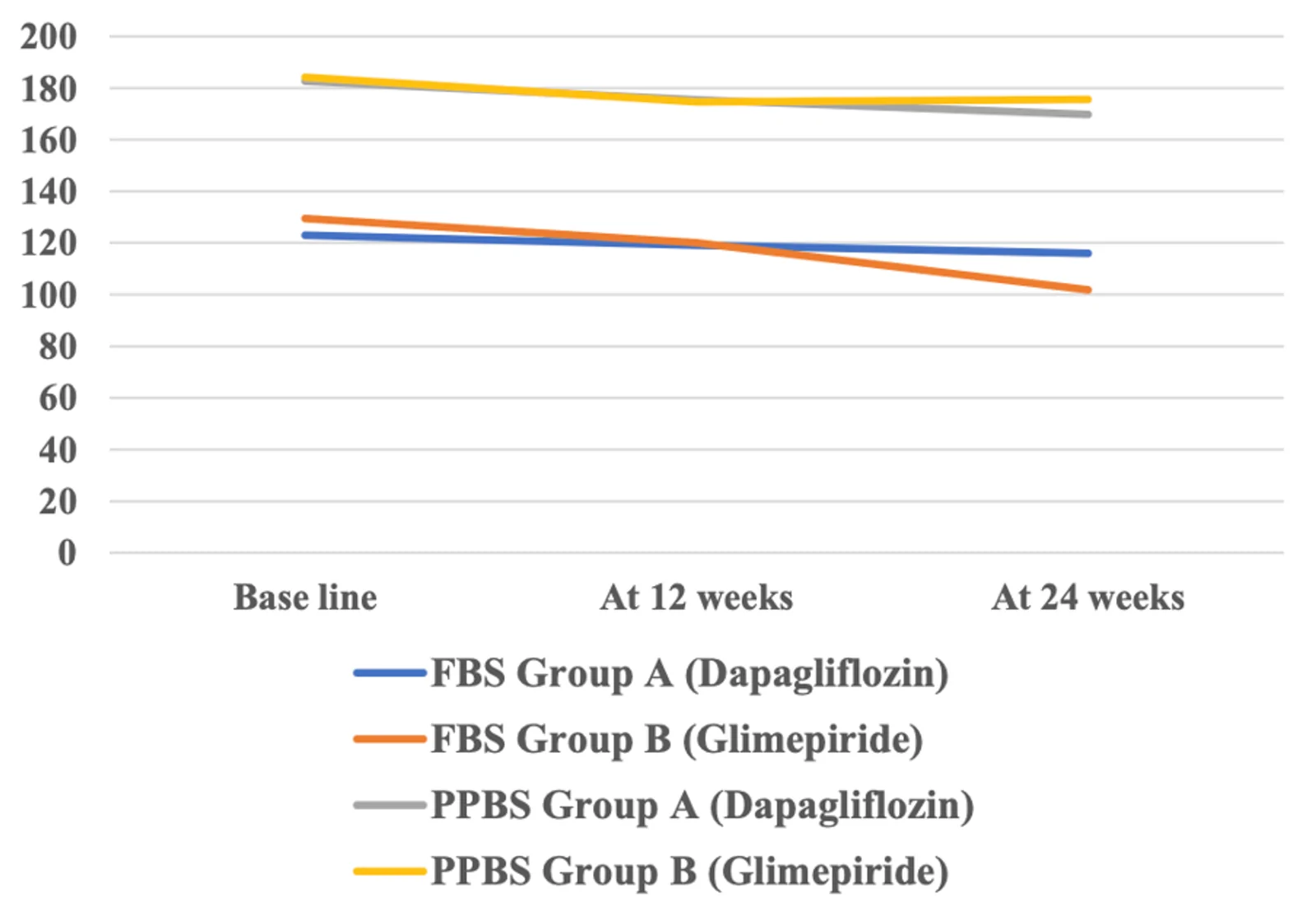
Trends in fasting blood sugar (FBS) and postprandial blood sugar (PPBS) over the 24-week follow-up period The figure illustrates the changes in mean fasting blood sugar (FBS) and postprandial blood sugar (PPBS) levels in the dapagliflozin plus metformin (Group A) and glimepiride plus metformin (Group B) groups at baseline, 12 weeks, and 24 weeks. FBS and PPBS values are expressed as mean blood glucose concentrations (mg/dL).

The trends in FBS and PPBS over the 24-week follow-up period are illustrated in Figure 2.

Between-Group Comparison

Between-group comparisons of FBS, PPBS, and glycated HbA1c at baseline, 12 weeks, and 24 weeks are presented in Table 5. At baseline, the mean FBS was significantly higher in the glimepiride plus metformin group than in the dapagliflozin plus metformin group (127.94±21.09 mg/dL vs. 122.98±20.14 mg/dL; p=0.09). However, no significant between-group differences were observed in PPBS values (p=0.86) or glycated hemoglobin (HbA1c) (p=0.88).

**Table 5:** Comparison of glycemic parameters between treatment groups The table presents the between-group comparison of fasting blood sugar (FBS), postprandial blood sugar (PPBS), and glycated hemoglobin (HbA1c) at baseline, 12 weeks, and 24 weeks in the dapagliflozin plus metformin and glimepiride plus metformin groups. Data are presented as mean±standard deviation (SD). P values represent between-group comparisons at each study visit; *Indicates statistical significance at p<0.05.

Visit	Parameter	Dapagliflozin+Metformin	Glimepiride+Metformin	p-value
Baseline	FBS (mg/dL)	122.98±20.14	127.94±21.09	0.09
	PPBS (mg/dL)	182.65±10.29	184.18±15.42	0.86
	HbA1c (%)	9.13±0.57	9.09±0.62	0.88
12 Weeks	FBS (mg/dL)	119.05±11.77	120.03±10.57	0.56
	PPBS (mg/dL)	175.36±13.96	175.59±31.09	0.139
	HbA1c (%)	8.33±0.63	8.42±0.68	0.41
24 Weeks	FBS (mg/dL)	115.98±15.09	101.82±20.65	<0.001*
	PPBS (mg/dL)	169.75±18.85	174.60±16.37	0.45
	HbA1c (%)	7.60±0.64	8.00±0.68	0.01*

At the 12-week follow-up, both groups demonstrated comparable glycemic control, with no statistically significant between-group differences in FBS (p=0.56), PPBS (p=0.139), or HbA1c (p=0.41).

By week 24, significant differences were observed between the treatment groups. The glimepiride plus metformin group achieved significantly lower FBS than the dapagliflozin plus metformin group (101.82±20.65 mg/dL vs. 115.98±15.09 mg/dL; p<0.001). In contrast, HbA1c was significantly lower in the dapagliflozin plus metformin group than in the glimepiride plus metformin group (7.60±0.64% vs. 8.00±0.68%; p=0.01), indicating better long-term glycemic control. No statistically significant between-group difference was observed in PPBS at week 24 (p=0.45).

**Figure 3:**
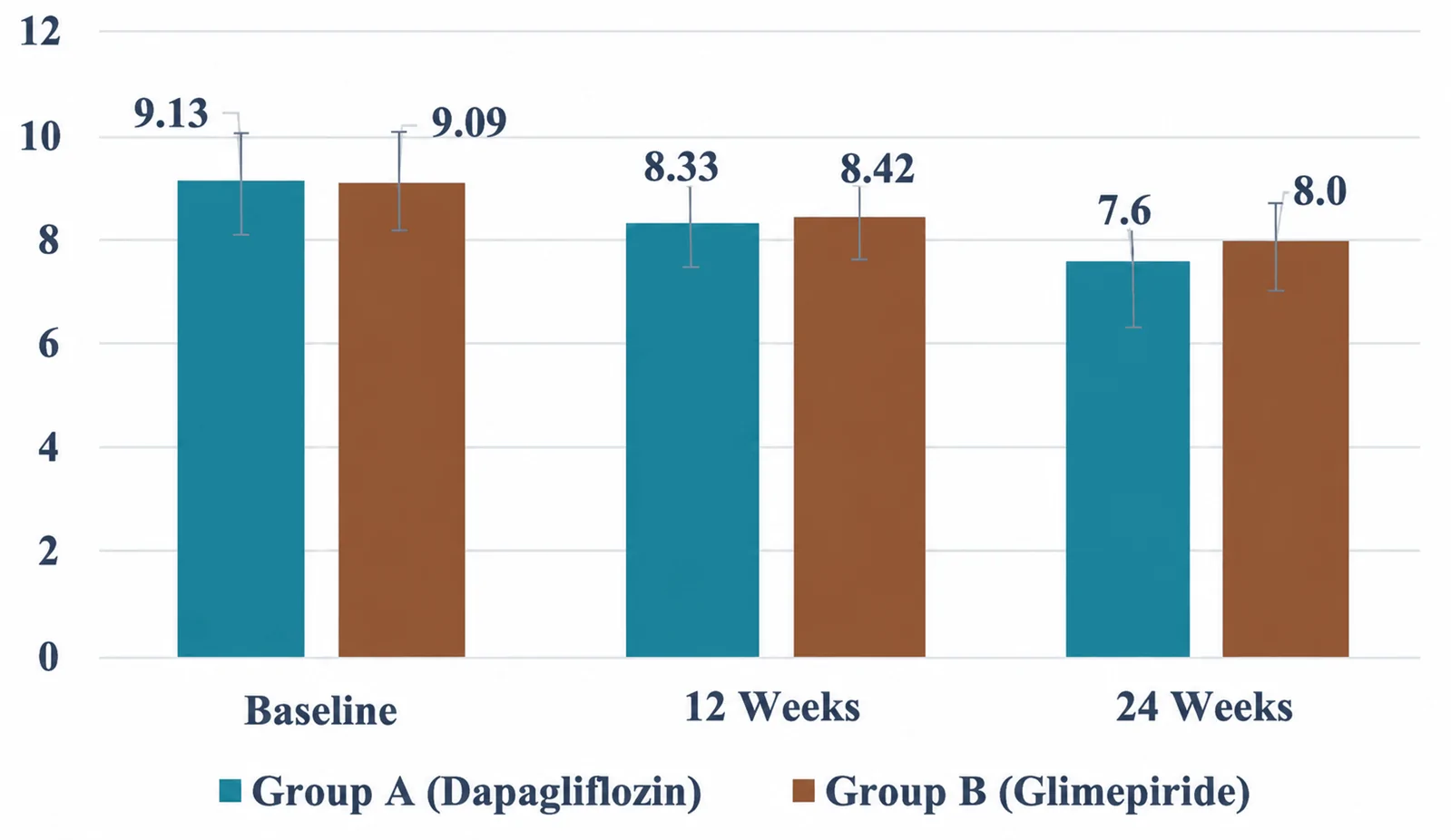
Comparison of glycated hemoglobin (HbA1c) levels between treatment groups over the 24-week follow-up period The figure compares the mean glycated hemoglobin (HbA1c) levels in the dapagliflozin plus metformin (Group A) and glimepiride plus metformin (Group B) groups at baseline, 12 weeks, and 24 weeks. Error bars represent the standard deviation (SD). HbA1c values are expressed as percentages (%).

The comparison of HbA1c levels between the dapagliflozin plus metformin and glimepiride plus metformin groups over the 24-week follow-up period is illustrated in Figure 3.

Body weight

A significant difference in body weight was observed between the two treatment groups over the 24-week follow-up period. The comparison of body weight between the dapagliflozin plus metformin and glimepiride plus metformin groups at baseline, 12 weeks, and 24 weeks is presented in Table 6. Participants receiving dapagliflozin plus metformin demonstrated a progressive reduction in body weight, whereas those receiving glimepiride plus metformin showed a gradual increase.

**Table 6:** Comparison of body weight between treatment groups during the 24-week follow-up period The table presents the between-group comparison of mean body weight in the dapagliflozin plus metformin and glimepiride plus metformin groups at baseline, 12 weeks, and 24 weeks. Data are presented as mean±standard deviation (SD). P values represent between-group comparisons at each study visit; *Indicates statistical significance at p<0.05.

Visit	Dapagliflozin+Metformin (n=85)	Glimepiride+Metformin (n=85)	p-value
Baseline	83.53±7.40	82.02±6.59	0.24
12 Weeks	81.56±7.41	83.33±6.57	0.08
24 Weeks	79.57±7.45	84.71±6.82	<0.001*

Within the dapagliflozin group, mean body weight decreased significantly from 83.53±7.40 kg at baseline to 81.56±7.41 kg at 12 weeks and 79.57±7.45 kg at 24 weeks (p<0.001). In contrast, the glimepiride group showed a significant increase in mean body weight from 82.02±6.59 kg at baseline to 83.33±6.57 kg at 12 weeks and 84.71±6.82 kg at 24 weeks (p<0.001).

Between-group comparisons showed no significant differences in mean body weight at baseline (p=0.24) or at 12 weeks (p=0.08). However, at 24 weeks, mean body weight was significantly lower in the dapagliflozin group than in the glimepiride group (79.57±7.45 kg vs. 84.71±6.82 kg, p<0.001).

**Figure 4:**
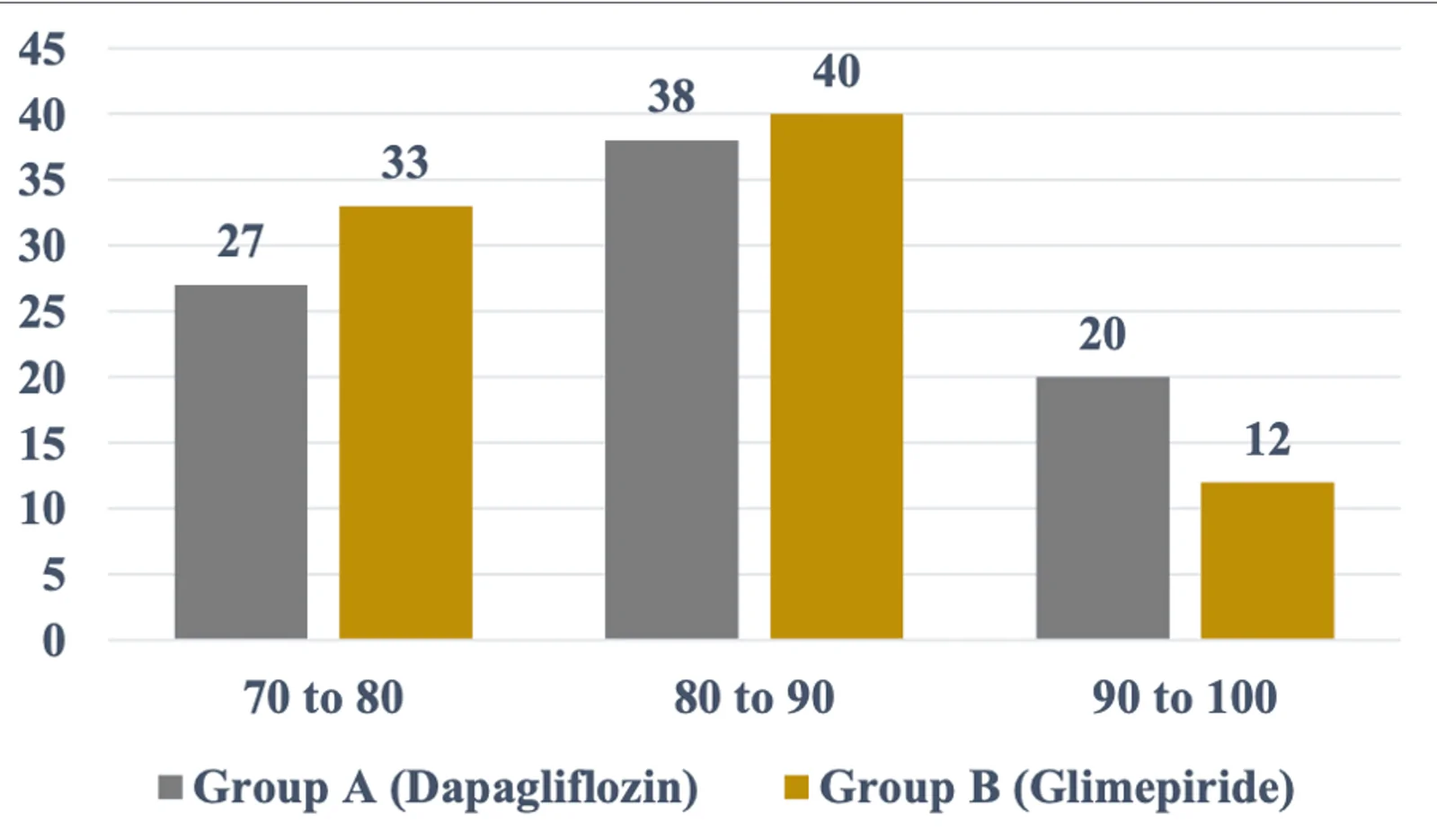
Baseline body weight distribution of participants in the dapagliflozin plus metformin and glimepiride plus metformin groups The figure illustrates the distribution of study participants according to baseline body weight categories in the dapagliflozin plus metformin (Group A) and glimepiride plus metformin (Group B) groups. The majority of participants in both groups had a baseline body weight between 80 and 90 kg.

The baseline body weight distribution of participants in both treatment groups is shown in Figure 4.

Blood pressure

The comparison of systolic blood pressure (SBP) and diastolic blood pressure (DBP) between the dapagliflozin plus metformin and glimepiride plus metformin groups at baseline, 12 weeks, and 24 weeks is presented in Table 7. Both treatment groups had comparable SBP and DBP at baseline. During the 24-week follow-up period, participants receiving dapagliflozin plus metformin demonstrated progressive reductions in both SBP and DBP, whereas those receiving glimepiride plus metformin showed gradual increases.

**Table 7:** Comparison of systolic and diastolic blood pressure between treatment groups during the 24-week follow-up period The table presents the between-group comparison of systolic blood pressure (SBP) and diastolic blood pressure (DBP) in the dapagliflozin plus metformin and glimepiride plus metformin groups at baseline, 12 weeks, and 24 weeks. Data are presented as mean±standard deviation (SD). P values represent between-group comparisons at each study visit; *Indicates statistical significance at p<0.05.

Visit	Parameter	Dapagliflozin+Metformin	Glimepiride+Metformin	p-value
Baseline	SBP (mmHg)	140.64±6.12	139.56±6.41	0.27
	DBP (mmHg)	85.15±3.27	84.45±3.12	0.15
12 Weeks	SBP (mmHg)	137.93±6.31	140.75±6.24	0.04*
	DBP (mmHg)	83.48±3.26	85.47±3.01	0.02*
24 Weeks	SBP (mmHg)	134.44±6.40	141.82±6.58	<0.001*
	DBP (mmHg)	81.11±3.38	86.74±3.22	<0.001*

At baseline, the mean SBP was 140.64±6.12 mmHg in the dapagliflozin group and 139.56±6.41 mmHg in the glimepiride group (p=0.27). The corresponding mean DBP values were 85.15±3.27 mmHg and 84.45±3.12 mmHg, respectively (p=0.15).

At 12 weeks, both SBP and DBP were significantly lower in the dapagliflozin group than in the glimepiride group (SBP: 137.93±6.31 vs. 140.75±6.24 mmHg, p=0.04; DBP: 83.48±3.26 vs. 85.47±3.01 mmHg, p=0.02).

By 24 weeks, these differences became more pronounced. The dapagliflozin group had significantly lower mean SBP (134.44±6.40 mmHg vs. 141.82±6.58 mmHg, p<0.001) and mean DBP (81.11±3.38 mmHg vs. 86.74±3.22 mmHg, p<0.001) than the glimepiride group.

**Figure 5:**
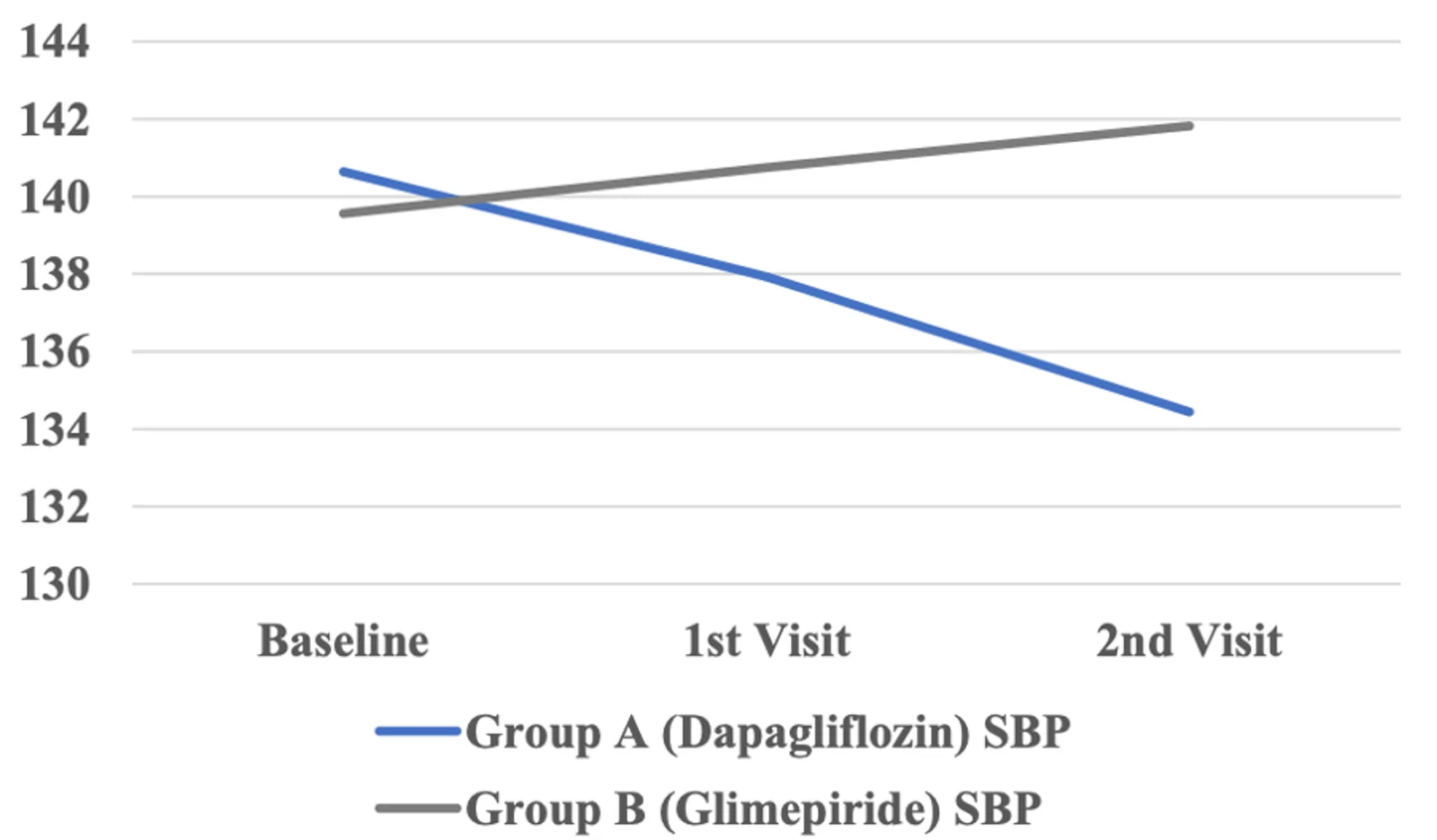
Comparison of systolic blood pressure (SBP) between treatment groups during the 24-week follow-up period The figure illustrates the changes in mean systolic blood pressure (SBP) in the dapagliflozin plus metformin (Group A) and glimepiride plus metformin (Group B) groups at baseline, 12 weeks (first visit), and 24 weeks (second visit). SBP values are expressed in millimeters of mercury (mmHg).

The trends in SBP during the 24-week follow-up period are illustrated in Figure 5.

**Figure 6:**
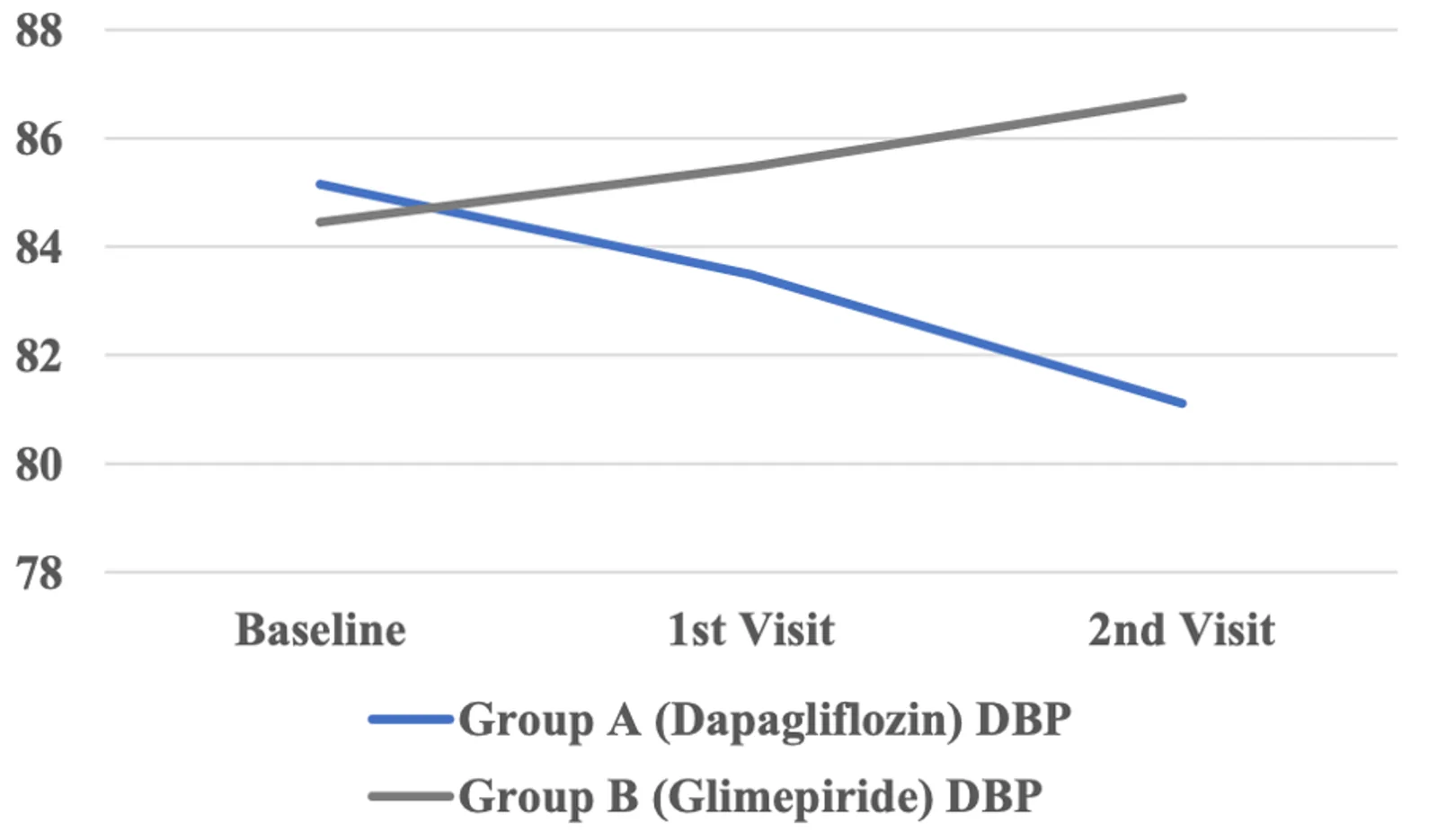
Comparison of diastolic blood pressure (DBP) between treatment groups during the 24-week follow-up period The figure illustrates the changes in mean diastolic blood pressure (DBP) in the dapagliflozin plus metformin (Group A) and glimepiride plus metformin (Group B) groups at baseline, 12 weeks (first visit), and 24 weeks (second visit). DBP values are expressed in millimeters of mercury (mmHg).

The corresponding trends in DBP during the 24-week follow-up period are illustrated in Figure 6.

Adverse events

The overall incidence of adverse events in the dapagliflozin plus metformin and glimepiride plus metformin groups is presented in Table 8. Overall, adverse events were reported in 30 participants (35.3%) in the dapagliflozin plus metformin group and 17 participants (20.0%) in the glimepiride plus metformin group. The overall incidence of adverse events was significantly higher in the dapagliflozin group than in the glimepiride group (p=0.04).

**Table 8:** Overall incidence of adverse events in the treatment groups The table presents the overall incidence of adverse events in participants receiving dapagliflozin plus metformin and glimepiride plus metformin during the 24-week follow-up period. Data are presented as number (percentage). The p value represents the between-group comparison of the overall incidence of adverse events; *Indicates statistical significance at p<0.05.

Adverse Events	Dapagliflozin+Metformin (n=85)	Glimepiride+Metformin (n=85)	p-value
Yes	30 (35.3%)	17 (20.0%)	0.04*
No	55 (64.7%)	68 (80.0%)	

**Figure 7:**
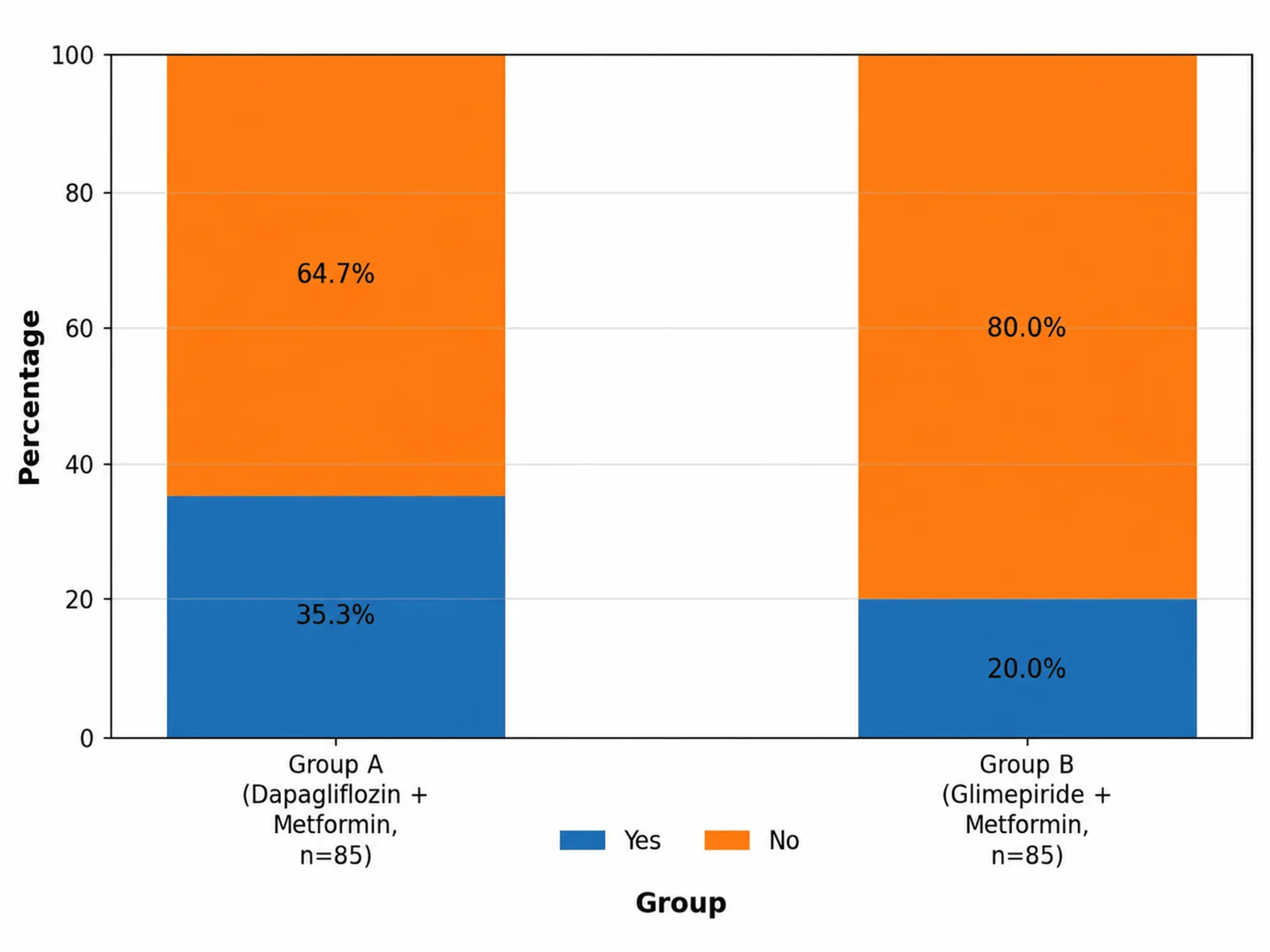
Overall incidence of adverse events in the dapagliflozin plus metformin and glimepiride plus metformin groups The figure illustrates the proportion of participants who experienced at least one adverse event during the 24-week follow-up period in the dapagliflozin plus metformin (Group A) and glimepiride plus metformin (Group B) groups. The overall incidence of adverse events was higher in the dapagliflozin group than in the glimepiride group.

The overall distribution of adverse events between the two treatment groups is illustrated in Figure 7. 

The distribution of specific adverse drug reactions is presented in Table 9. The frequency of specific adverse drug reactions varied between the two treatment groups. Fatigue (16.5%), polyuria (15.3%), headache (12.9%), nasal congestion (10.6%), and genital infections (8.2%) were reported more frequently among participants receiving dapagliflozin plus metformin. In contrast, tremors (14.1%), hypoglycemia (7.1%), weight gain (4.7%), and sweating (3.5%) were reported more frequently among participants receiving glimepiride plus metformin.

**Table 9:** Distribution of adverse drug reactions in the dapagliflozin plus metformin and glimepiride plus metformin groups The table summarizes the frequency of specific adverse drug reactions reported during the 24-week follow-up period in participants receiving dapagliflozin plus metformin and glimepiride plus metformin. Data are presented as number (percentage). Participants may have experienced more than one adverse drug reaction; therefore, the total number of adverse drug reactions may exceed the number of participants reporting adverse events.

Adverse Drug Reaction	Dapagliflozin+Metformin n (%)	Glimepiride+Metformin n (%)
Fatigue	14 (16.5)	8 (9.4)
Headache	11 (12.9)	8 (9.4)
Polyuria	13 (15.3)	5 (5.9)
Nasal congestion	9 (10.6)	3 (3.5)
Hypoglycemia	0 (0.0)	6 (7.1)
Weight gain	0 (0.0)	4 (4.7)
Tremors	0 (0.0)	12 (14.1)
Genital infection	7 (8.2)	4 (4.7)
Sweating	0 (0.0)	3 (3.5)
Abdominal pain	2 (2.4)	1 (1.2)

**Figure 8:**
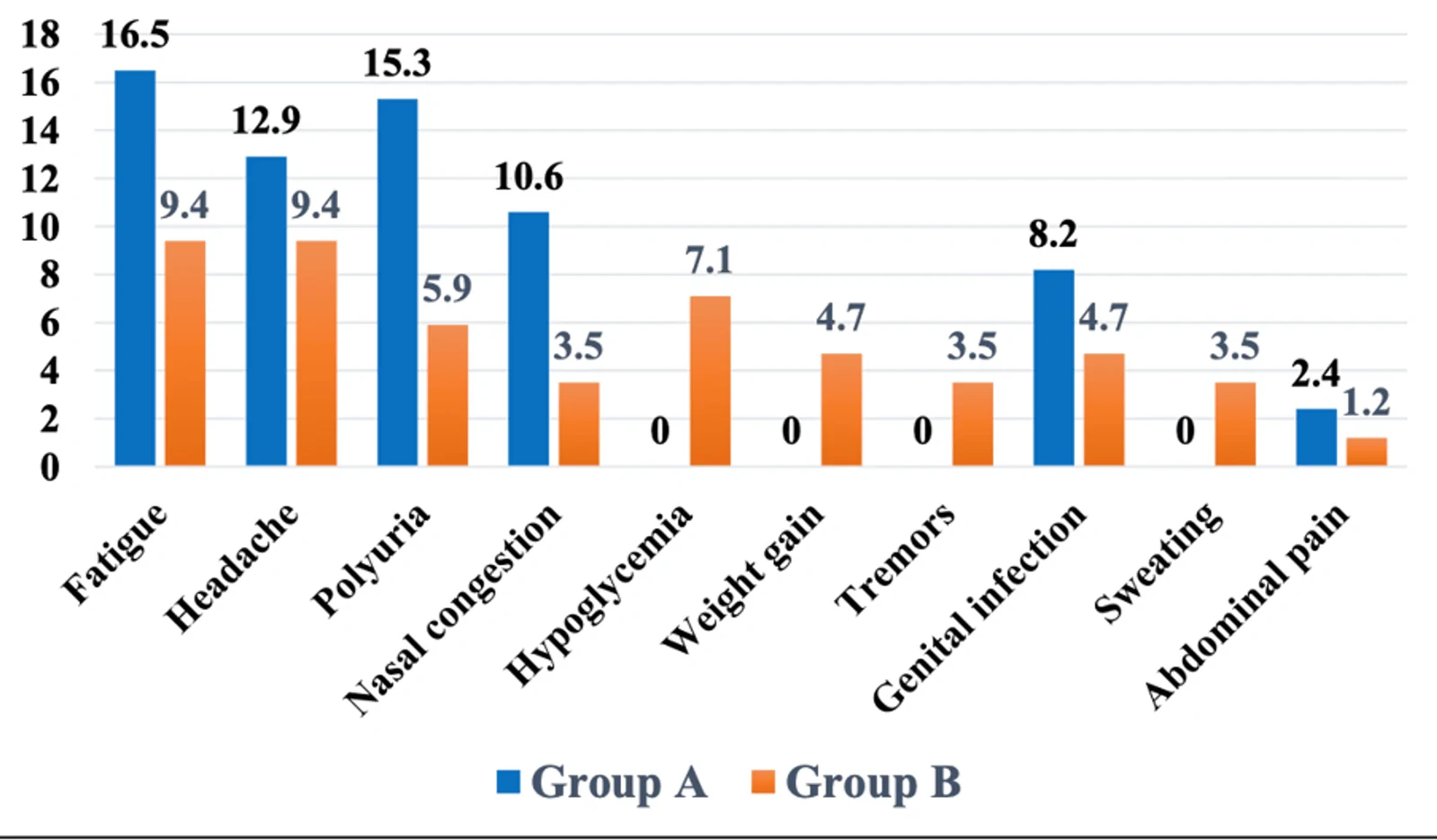
Distribution of adverse drug reactions in the dapagliflozin plus metformin and glimepiride plus metformin groups The figure compares the frequency of specific adverse drug reactions reported during the 24-week follow-up period in participants receiving dapagliflozin plus metformin (Group A) and glimepiride plus metformin (Group B). Values represent the percentage of participants reporting each adverse drug reaction. Participants may have experienced more than one adverse drug reaction.

The distribution of specific adverse drug reactions across the two treatment groups is illustrated in Figure 8. 

## Discussion

The present prospective comparative study evaluated the efficacy and safety of dapagliflozin and glimepiride as add-on therapy to metformin in patients with inadequately controlled T2DM. Both treatment groups demonstrated significant improvements in glycemic control over the 24-week follow-up period, confirming the effectiveness of combination therapy in patients whose blood glucose levels remain uncontrolled with metformin alone. However, dapagliflozin was associated with superior improvement in HbA1c, significant reductions in body weight and blood pressure, and a lower incidence of hypoglycemia, whereas glimepiride produced greater reductions in FBS but was associated with weight gain and more frequent hypoglycemic episodes. These findings support the growing body of evidence suggesting that treatment selection in T2DM should extend beyond glycemic efficacy and consider patient-specific factors such as cardiovascular risk, body weight, adverse effects, and long-term metabolic benefits [7,8].

The baseline demographic characteristics of both treatment groups were comparable with respect to age, sex distribution, baseline glycemic parameters, body weight, and blood pressure, indicating that the observed differences at the end of the study were primarily attributable to the therapeutic interventions rather than baseline imbalances. Similar baseline comparability has been reported in previous randomized controlled trials comparing SGLT2 inhibitors with sulfonylureas, including the studies by Nauck et al. and Park et al., thereby supporting the internal validity of the present study [17,19].

Although FBS showed a numerical reduction in the dapagliflozin group over 24 weeks, this change did not reach statistical significance. In contrast, the glimepiride group demonstrated a statistically significant within-group reduction in FBS. The reduction in HbA1c was significantly greater in the dapagliflozin group, whereas glimepiride achieved a greater reduction in FBS. These findings are consistent with previous clinical trials demonstrating that SGLT2 inhibitors provide glycemic efficacy comparable to sulfonylureas while offering additional metabolic benefits. The glucose-lowering effect of dapagliflozin results from inhibition of sodium-glucose cotransporter-2 in the proximal renal tubules, leading to increased urinary glucose excretion independent of pancreatic β-cell function or insulin secretion. In contrast, glimepiride enhances insulin secretion by closing ATP-sensitive potassium channels on pancreatic β-cells, making its efficacy dependent on residual β-cell function [10,12,21].

The greater reduction in HbA1c observed with dapagliflozin in the present study is in agreement with the randomized non-inferiority trial conducted by Nauck et al., which demonstrated that dapagliflozin provided glycemic control comparable to glipizide while significantly reducing body weight and minimizing hypoglycemia over 52 weeks. Similarly, Goring et al. reported in their network meta-analysis that dapagliflozin combined with metformin achieved glycemic control comparable to sulfonylureas but demonstrated a more favorable safety profile and superior weight reduction [18]. The BEYOND study conducted by Park et al. further confirmed that dapagliflozin produced significant reductions in body fat mass, visceral adiposity, and body weight compared with glimepiride despite achieving similar glycemic control [17,19].

A notable finding of the present study was the significant reduction in body weight among patients receiving dapagliflozin, whereas patients receiving glimepiride experienced modest weight gain. Weight reduction associated with SGLT2 inhibitors is primarily attributed to urinary glucose excretion, resulting in caloric loss, together with mild osmotic diuresis and improved insulin sensitivity. In contrast, sulfonylureas stimulate endogenous insulin secretion, promoting lipogenesis and weight gain. The observed findings are consistent with previous randomized trials and meta-analyses that have consistently demonstrated clinically meaningful weight reduction with dapagliflozin compared with sulfonylureas [14,15,22].

Patients receiving dapagliflozin also demonstrated significant reductions in systolic and diastolic blood pressure. This effect is likely attributable to osmotic diuresis, natriuresis, plasma volume contraction, and modest weight reduction associated with SGLT2 inhibition. Similar blood pressure reductions have been consistently reported in major cardiovascular outcome trials, including EMPA-REG OUTCOME (Empagliflozin Cardiovascular Outcome Event Trial in Type 2 Diabetes Mellitus Patients - Removing Excess Glucose), DECLARE-TIMI 58 (Dapagliflozin Effect on Cardiovascular Events - Thrombolysis in Myocardial Infarction 58), and DAPA-HF (Dapagliflozin and Prevention of Adverse Outcomes in Chronic Heart Failure). These additional cardiometabolic benefits have substantially expanded the clinical role of SGLT2 inhibitors beyond glycemic control and have resulted in their recommendation for patients with cardiovascular disease, heart failure, and chronic kidney disease [13-16].

The safety findings of the present study were also consistent with the known pharmacological profiles of both drug classes. Although overall adverse events were more frequently observed in the dapagliflozin group, these were predominantly mild events such as polyuria and genital infections resulting from glucosuria. Conversely, symptomatic hypoglycemia and tremors occurred more frequently among patients receiving glimepiride because of its insulin secretagogue mechanism. Similar safety profiles have been consistently reported in previous randomized trials and systematic reviews evaluating dapagliflozin and sulfonylureas [11,17,21,23].

The findings of the present study have important clinical implications, particularly in resource-limited settings such as India, where treatment affordability often influences prescribing practices. Although glimepiride remains an effective and inexpensive option for improving glycemic control, dapagliflozin offers several additional advantages, including weight reduction, lower hypoglycemia risk, blood pressure reduction, and proven cardiovascular and renal benefits. Consequently, individualized treatment decisions should consider not only glycemic efficacy but also patient comorbidities, cardiovascular risk profile, body weight, renal function, susceptibility to hypoglycemia, and treatment affordability.

The strengths of the present study include its prospective design, direct comparison of two commonly prescribed second-line oral antidiabetic agents, and assessment of multiple clinically relevant outcomes, including glycemic parameters, body weight, blood pressure, and adverse drug reactions.

However, several limitations should be acknowledged. The study was conducted at a single tertiary care center with a relatively modest sample size and a follow-up duration of only 24 weeks, limiting the assessment of long-term cardiovascular and renal outcomes. As this was a prospective, nonrandomized observational study with physician-directed treatment allocation, confounding by indication and residual confounding cannot be excluded, and causal inferences cannot be established. In addition, treatment allocation was not blinded, which may have introduced observer bias. Larger multicenter randomized studies with longer follow-up are warranted to validate these findings and assess long-term clinical outcomes.

Overall, the present study demonstrates that both dapagliflozin and glimepiride are effective add-on therapies to metformin for patients with inadequately controlled T2DM. However, dapagliflozin offers clinically meaningful advantages beyond glycemic control, particularly with respect to body weight, blood pressure, and hypoglycemia risk, supporting its role as an effective therapeutic option for individualized diabetes management.

## Conclusions

Both dapagliflozin and glimepiride were effective as add-on therapies to metformin in improving glycemic control in participants with inadequately controlled T2DM. During the 24-week study period, both treatment groups demonstrated improvements in FBS, PPBS, and HbA1c. Dapagliflozin was associated with greater improvement in HbA1c, significant reductions in body weight and blood pressure, and a lower incidence of hypoglycemia compared with glimepiride. Conversely, glimepiride produced greater reductions in FBS but was associated with weight gain and a higher frequency of hypoglycemic events and remains an effective and economical treatment option, particularly in resource-constrained settings where medication cost may influence therapeutic decisions.

Overall, treatment selection should be individualized according to participant characteristics, comorbidities, risk of adverse events, and therapeutic priorities. Further large-scale, multicenter studies with longer follow-up are needed to evaluate the long-term cardiovascular, renal, and safety outcomes of dapagliflozin compared with sulfonylureas across diverse participant populations.
